# Biocompatible Chitosan Nanobubbles for Ultrasound-Mediated Targeted Delivery of Doxorubicin

**DOI:** 10.1186/s11671-019-2853-x

**Published:** 2019-01-16

**Authors:** Xiaoying Zhou, Lu Guo, Dandan Shi, Sujuan Duan, Jie Li

**Affiliations:** Department of Ultrasound, Qilu Hospital of Shandong University, West Wenhua Road, Jinan, Shandong China

**Keywords:** Nanobubbles, Targeted drug delivery, Biocompatible, Ultrasound

## Abstract

Ultrasound-targeted delivery of nanobubbles (NBs) has become a promising strategy for noninvasive drug delivery. The biosafety and drug-transporting ability of NBs have been a research hotspot, especially regarding chitosan NBs due to their biocompatibility and high biosafety. Since the drug-carrying capacity of chitosan NBs and the performance of ultrasound-assisted drug delivery remain unclear, the aim of this study was to synthesize doxorubicin hydrochloride (DOX)-loaded biocompatible chitosan NBs and assess their drug delivery capacity. In this study, the size distribution of chitosan NBs was measured by dynamic light scattering, while their drug-loading capacity and ultrasound-mediated DOX release were determined by a UV spectrophotometer. In addition, a clinical ultrasound imaging system was used to evaluate the ability of chitosan NBs to achieve imaging enhancement, while the biosafety profile of free chitosan NBs was evaluated by a cytotoxicity assay in MCF-7 cells. Furthermore, NB-mediated DOX uptake and the apoptosis of Michigan Cancer Foundation-7 (MCF-7) cells were measured by flow cytometry. The results showed that the DOX-loaded NBs (DOX-NBs) exhibited excellent drug-loading ability as well as the ability to achieve ultrasound enhancement. Ultrasound (US) irradiation promoted the release of DOX from DOX-NBs in vitro. Furthermore, DOX-NBs effectively delivered DOX into mammalian cancer cells. In conclusion, biocompatible chitosan NBs are suitable for ultrasound-targeted DOX delivery and are thus a promising strategy for noninvasive and targeted drug delivery worthy of further investigation.

## Background

Chemotherapy is currently used as the primary treatment modality for malignant neoplasms and substantially improves the survival rate of cancer patients. Nevertheless, the efficacy of chemotherapeutic drugs is restricted by their adverse side effects, such as systemic toxicity [1]. Local delivery of chemotherapeutic drugs may reduce their toxicity by increasing their therapeutic dose at targeted sites and by decreasing the plasma levels of circulating drugs. Due to its noninvasiveness and targetability, ultrasound-targeted nano/microbubble destruction (UTN/MD) has been widely used as an effective drug delivery system.

Compared to traditional microbubbles, nanosized particles can cross the capillary wall more easily and hence can be delivered to the target site more efficiently. NBs have been used in the study of targeted therapy, such as 5-fluorouracil-loaded NBs tested for use in hepatocellular carcinoma [2]. Shen et al. recently used ultrasound-mediated NBs to deliver resveratrol to nucleus pulposus cells [3], and NBs have also been used in the treatment of breast cancer [4].

The nanobubbles (NBs) used in UTN/MD are usually composed of a gas core and a stabilized shell. Lipids, surfactants, polymers, or other materials are used in the composition of the shell. Different types of NBs have been made in previous studies. However, many of the chemicals used to form NBs or nanoparticles pose a potential threat to the human body. Consequently, the transport of some nanoparticles has brought unsatisfactory therapeutic efficacy and toxicity in normal tissues and cells [5]. Chemical agents, such as Tween 80 and glutaraldehyde, have high toxicity and pose mutagenic risks, thus restricting their clinical applications [6, 7]. PLA, another material used in NBs, may cause clinical side effects in some cases [8]. In this context, it is important to consider the biocompatibility and safety of the materials used to assemble NBs.

The polysaccharide chitosan has attracted attention due to its natural origin, biodegradability, biocompatibility, exceptionally low immunogenicity, antibacterial activity, and practicality [9, 10]. Chitosan is the N-deacetylated derivative of chitin, which is one of the most abundant biological materials on earth [11]. In addition, a previous study showed that, in the presence of IFN-γ, water-soluble chitosan oligomers can activate macrophages to kill cancer cells [12]. Therefore, chitosan itself has both direct and indirect antitumor effects, making it more suitable as a carrier for anticancer drugs. The other materials we used in our NBs were lecithin and palmitic acid, which are excellent candidates for use in NBs [13]. Palmitic acid is one of the most abundant of the saturated 14-, 16-, and 18-carbon fatty acids and is normally synthesized by acetyl-CoA and possesses low toxicity and high biocompatibility [14]. Lecithin is a native surfactant mainly derived from soybeans [15]. Previous research has shown that soy lecithin exhibits health benefits because of its hypocholesterolemic properties. For example, soy lecithin is helpful in reducing the risk of cardiovascular diseases, while purified soy could be used for encapsulating nisin [16, 17]. In this study, we used the above materials to make biocompatible NBs. With the assistance of ultrasound for delivery, doxorubicin hydrochloride (DOX) was used as a model drug to test the drug-loading capacity of the novel, biogenic chitosan NBs, which were functionalized prior to evaluation in human Michigan Cancer Foundation-7 (MCF-7) breast cancer cells. In addition, the antitumor effects of DOX-NBs were also assessed following UTN/MD.

## Materials and Methods

### Materials

The NBs described in this study were constructed using perfluoropropane (C3F8, R&D Center for Specialty Gases at the Research Institute of Physical and Chemical Engineering of Nuclear Industry, Beijing, China) as the core and a chitosan coating as the shell. In addition, Epikuron 200 (soy lecithin containing 95% of dipalmitoylphosphatidylcholine, Lukas Meyer, Hamburg, Germany), ethanol (analytical grade, Hushi, China), doxorubicin hydrochloride (Sigma-Aldrich, Missouri, USA), chitosan (100~300 kD, Bozhihuili, Qingdao, China), and palmitic acid (JINDU, Shanghai, China) were also used in this study. Pluronic F68 was purchased from Sigma-Aldrich (St. Louis, MO, USA).

### Cell Line

MCF-7 human breast carcinoma cell line was obtained from the American Type Culture Collection (Rockville, MD, USA) and cultured in Dulbecco’s modified Eagle’s medium (DMEM) supplemented with 10% heat-inactivated fetal bovine serum (FBS) (Gibco, Carlsbad, CA, USA). The cells were cultured under 37 °C, 5% CO_2_, and 95% humidity. Cells in the logarithmic growth phase were harvested for experiments.

### Preparation of DOX-Loaded Chitosan NBs

We made NBs according to previously described methods [18, 19]. Medium molecular weight chitosan (100~300 kD) was used for the shells of the DOX-NBs, and perfluoropropane was used for the core. To prepare the DOX-chitosan solution, the appropriate dose of DOX was dissolved in ultrapure water and 2 ml of DOX solution (1 mg/mL) was added to the chitosan water solution by mixing with a vortex mixer for 5 s. The DOX-chitosan solution was incubated for 1 h at 65 °C. Separately, an ethanol solution containing Epikuron 200 was added to an aqueous palmitic acid solution. After adding the appropriate volume of ultrapure water, the palmitic acid-Epikuron 200 system was homogenized using a vortex mixer. Subsequently, the palmitic acid-Epikuron 200 system was divided into 1.5-mL Eppendorf tubes (EP tubes), and the air in the tube was replaced with perfluoropropane using a 10-mL syringe with a long fine needle. Each tube was oscillated for 120 s in a mechanical oscillator (Ag and Hg mixer, Xi’an, China). Next, all the liquid in the 1.5-mL EP tubes were poured into a centrifuge tube and combined with the DOX-chitosan solution in an ice bath. Subsequently, the mixture was incubated for 30 min at − 4 °C. Next, an aqueous solution of Pluronic F68 (0.01%, *w*/*w*), a stabilizing agent, was added to the above mixture while stirring. A dialysis purification step (ultrafiltration centrifuge tube, Millipore, 30 kDa) was then performed to remove any residual free DOX.

### Observation of the Physical Properties of the NBs

The suspension of DOX-NBs was diluted by adding an appropriate amount of PBS (phosphate-buffered saline). The shape of DOX-NBs was then observed and imaged under a fluorescence microscope equipped with a × 100 oil-immersion objective lens (OLYMPUS BX41, Olympus Corporation, Japan). The fluorescence images were assessed using a fluorescence microscope (Nikon TE2000-S, Japan). The DOX-NBs’ morphology was also observed by transmission electron microscopy (TEM) (JEOL, Tokyo, Japan). The diluted nanobubbles’ aqueous suspensions were sprayed on Formvar-coated copper grid and stained with 4% *w*/*v* uranyl acetate for 10 min. Then the samples were visualized and imaged using TEM. The size and surface zeta potential of the DOX-NBs were measured by a Delsa Nano C particle size and zeta potential analyzer (Beckmann Instruments, USA). All measurements were performed in triplicate to calculate the mean value.

### Stability of DOX-NBs

The size and morphology of DOX-NBs were measured over time to observe the stability of these nanobubbles. Some parts of the DOX-NBs were stored in refrigerator at 4 °C for 24 h or 48 h. The others were kept at room temperature for 6 h. Nanobubbles stability at 25 °C was also investigated in lyophilized human serum (Seronorm™ Human, Norway). For this purpose, 1 ml of the DOX-NBs aqueous suspension was added to 1 ml of the serum and incubated for 6 h at 25 °C. Then, all the DOX-NBs were measured by morphology analysis using optical microscopy to evaluate the integrity of their structures. The size of the NBs was measured by a Delsa Nano C particle size and zeta potential analyzer (Beckmann Instruments, USA).

### Determination of DOX-Loading Capacity of NBs

A standard curve of DOX concentration was prepared using serial DOX dilutions at concentrations of 0.025, 0.05, 0.1, and 0.2 mg/mL and measured using a UV spectrophotometer (UV-2450, SHIMADZU). Subsequently, DOX-loading efficiency was assessed at 480 nm using blank NBs as the control, and the DOX concentration in DOX-NBs was calculated based on the standard curve established above. To avoid the photodegradation of DOX during the purification and measurement process, all procedures were performed while protected from light. Subsequently, the suspension of DOX-NBs was freeze-dried for 1.5 days with a freeze-dryer at − 55 °C and under 0.080 mbar [20]. Freeze-dried DOX-NBs were then weighed to calculate the drug-loading capacity in terms of drug encapsulation efficiency (EE) as follows: EE = *A*/*B* × 100%, where *A* is the amount of DOX loaded in NBs, and *B* is the initial amount of DOX in the solution. The experiments were repeated three times.

### Ultrasound-Mediated DOX Release

The in vitro release kinetics of DOX from the DOX-NBs was determined in the presence and absence of ultrasound (US) by dialysis bag technique at 37 °C. The DOX-NBs were enclosed in a dialysis membrane (Spectra/Por, cutoff 12,000–14,000 Da), which was placed in a container of 100 ml PBS with shaking at 100 rpm. The US group was sonicated by US (VCX400, Sonics and Materials, USA; power density, 1.0 W/cm2; frequency, 20 kHz) for 40 s before testing [21]. DOX release was measured for up to 24 h, withdrawing 1 ml at each fixed time and replacing with 1 ml of fresh PBS. The concentrations of DOX in the external buffer were measured at 480 nm by a UV spectrophotometer. The release experiments were performed in triplicate.

### In Vitro Ultrasound Imaging (Time-Intensity Curve)

The US imaging and stability of DOX-NBs under ultrasound were verified in vitro on a clinical ultrasound scanner system (LOGIQ E9; GE, USA). The experiment was conducted with specific frequencies, transmission powers, and durations of exposure to ultrasound. Utilizing a previously developed method [19] (Fig. 5a), DOX-NBs achieved ultrasound enhancement. The ultrasound imaging stability of DOX-NBs was evaluated following their exposure to an ultrasound stimulus with a mechanical index (MI) of 0.10 and an imaging depth of 4.5 cm. All of the US images were analyzed offline with Image J. Subsequently, image analysis was conducted using the built-in software of LOGIQ E9 to calculate the gray-scale values of samples. For each of the 60-s clips, which were obtained from 0 to 15 min, motion correction was first performed for each frame and the decibel value was obtained. Each decibel value was plotted on a time-intensity curve to reflect the changes in contrast enhancement before and after ultrasonic irradiation. The peak intensity and duration of enhancement were expressed by a time-intensity curve. During the analysis, range-corrected backscatter values were obtained by subtracting the background signals corresponding to a water sample.

### Cytotoxicity Assay for Empty NBs

The biosafety of empty chitosan NBs was tested using a Cell Counting Kit-8 (CCK-8) assay kit (Sigma-Aldrich, USA). Prior to the assay, MCF-7 cells were plated at a density of 2 × 10^3^ cells/well in 96-well plates. The cells were subjected to treatment by varying concentrations of chitosan NBs and ultrasound conditions. The biosafety of chitosan NBs was evaluated by incubating MCF-7 cells at serial concentrations of NBs from 0 to 30%. Low intensity ultrasound stimulation equipment (US10, Cosmogamma Corporation, Italy) was used to perform ultrasound stimulation at a fixed frequency of 1 MHz, using a 70% duty cycle and a 100 Hz pulse rate. Each group processed with different irradiation time and different sound intensity. For safety considerations, ultrasound was used at an intensity of 0.5 W/cm^2^ or 1.0 W/cm^2^ with a pulse length of 30 or 60 s (Table 1). The depth, frequency, and other ultrasound conditions were kept consistent during all ultrasound experiments [22]. Following treatments, the cells were cultured in the 96-well plates for an additional 24 h. Subsequently, a maintenance medium containing 1% FCS was used to replace the drug-containing medium, and a CCK-8 solution was added into the plates according to the manufacturer’s instructions. Following an additional 2.5 h incubation at 37 °C, the spectrophotometric absorbance in each well was determined using a microplate reader (Bio-Rad, USA) at a wavelength of 450-nm.

**Table 1:** Ultrasonic conditions in different groups of the CCK-8 assay

	Group 1	Group 2	Group 3
Time (s)	30	60	30
Ultrasound intensity (W/cm^2^)	0.5	0.5	1.0

### Intracellular Drug Uptake In Vitro

The intracellular uptake of DOX was determined by flow cytometry (Beckman Coulter, Miami, USA). In brief, MCF-7 cells were plated into six-well plates at a density of 2.5 × 10^5^ cells/well in DMEM medium supplemented with 10% FBS. Following overnight culture, the medium was replaced by a culture medium containing DOX-NBs or free DOX at the same concentration, and the cells were treated with or without ultrasound. In consideration of cell viability and high sonoporation efficiency, a DOX-NBs concentration of 20% was chosen for the subsequent experiments, while the ultrasound treatment was set at an intensity of 0.5 W/cm^2^ with a pulse length of 30 or 60 s. Subsequently, after 1-h incubation, the cell medium was removed, and the cells were washed three times with fresh PBS to remove free and unbound DOX-NBs or DOX. The cells were then collected by centrifugation (5 min, 1000 rpm), resuspended in 500 μL of PBS prior to the intracellular uptake of DOX, and analyzed on a FACSCalibur flow cytometer. During the analysis, the gate was arbitrarily set for the detection of red fluorescence, and 10,000 cells were analyzed for each sample.

### The Effects of DOX-NBs on MCF-7 Cells In Vitro

CCK-8 assays and flow cytometry were used to conduct quantitative evaluation of MCF-7 cell proliferation and apoptosis following uptake of DOX. In brief, MCF-7 cells were plated and incubated in 96-well plates or 6-well plates and treated using the procedures described above. For the proliferation assay, the treated cells were incubated at 37 °C for 24 h, followed by CCK-8 staining for 2 h and absorbance reading on a microplate reader. DOX-induced apoptosis was determined by an Annexin V-APC assay as follows: after a six-hour treatment, the cells were stained by adding 0.5 μL V-APC (Sungene Biotech, Tianjin, China) into each well, and flow cytometry analysis (Beckman, Coulter, Fullerton, CA, USA) was used to quantify apoptotic cells.

### Statistical Analysis

All experiments were performed in triplicates and data were expressed as the mean. Statistical analyses were performed using SPSS Version 18.0. A *p* value < 0.05 was considered statistically significant.

## Results

### Physico-chemical Characterization of DOX-NBs

**Fig. 1:**
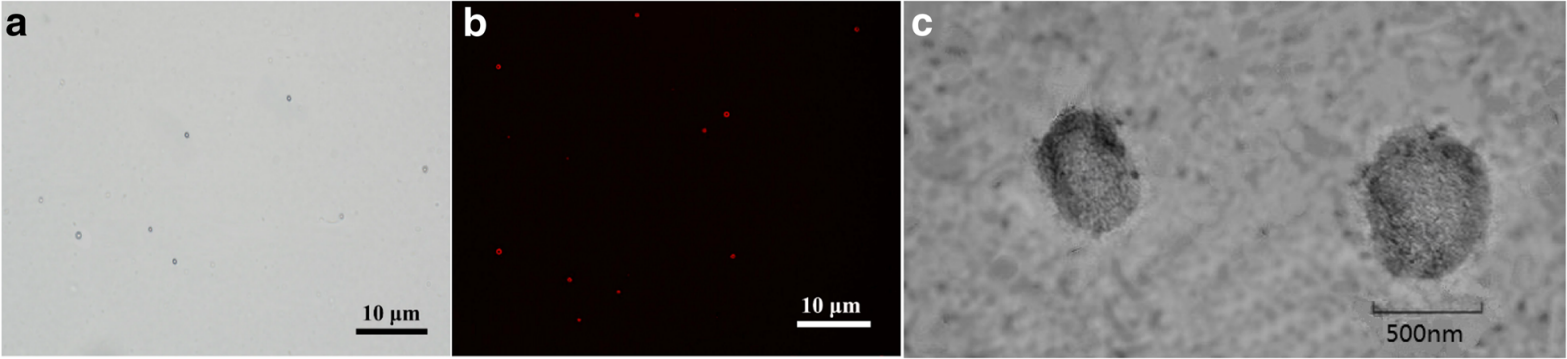
NBs observed under a light microscope (magnification × 1000) (**a**) and the fluorescence microscope image of DOX-loaded NBs (**b**) and TEM image of DOX-loaded NBs (**c**)

**Fig. 2:**
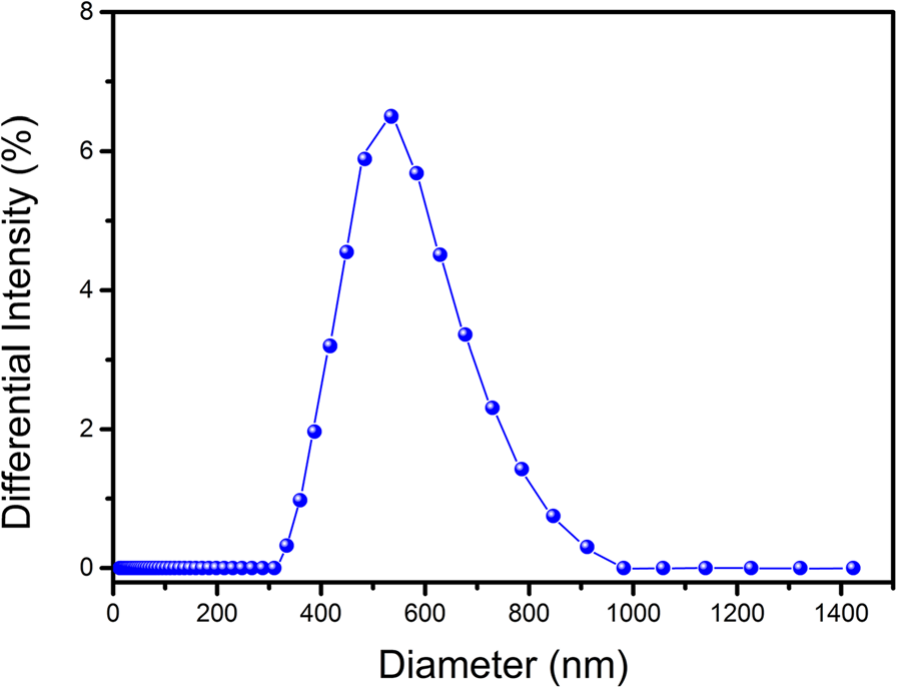
The size distribution of DOX-loaded NBs

The prepared NBs displayed a spherical morphology. Under an inverted microscope, imaging of NBs showed discrete and intact spherical outlines (Fig. 1a), which was consistent with the fluorescence microscope imaging of DOX-NBs (Fig. 1b). A representative TEM image of DOX-NBs’ solution is shown in Fig. 1c. The physical properties of the NBs were determined by the particle size and zeta potential analyzer. As shown in Fig. 2, the average diameter of DOX-NBs was 641 nm, P.I. 0.256. The zeta potential of DOX-NBs was + 67.12 ± 2.1 mV, which was sufficiently high to cause them to repel each other, aiding in the prevention of NB aggregation and supporting their long-term stability.

### Stability and Drug-Loading Efficiency of DOX-NBs

**Fig. 3:**
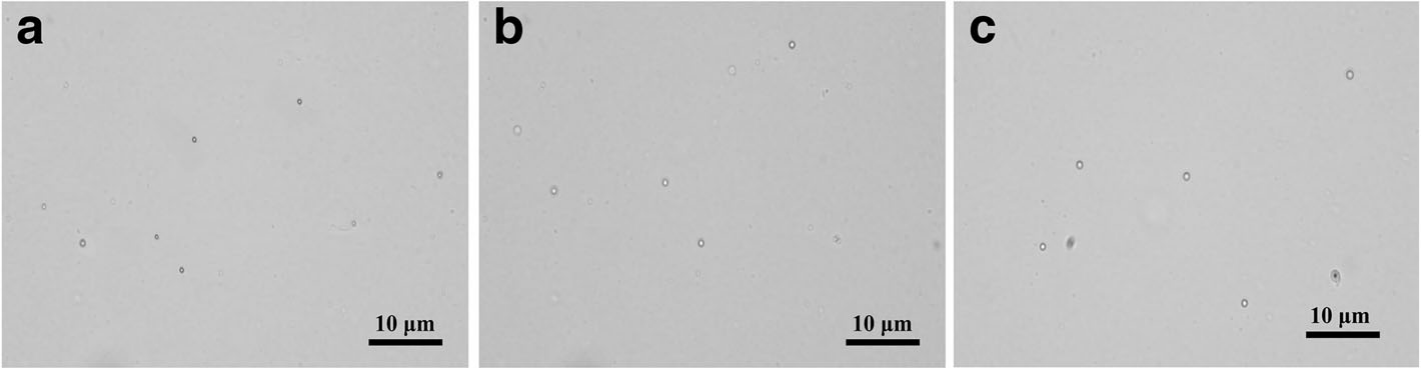
Optical images of DOX-NBs **a** at room temperature, **b** after 6 h at 25 °C in PBS, and **c** after 6 h at 25 °C in the serum

The DOX-NBs were stable in suspension for 48 h at 4 °C. After being stored at room temperature, the size of DOX-NBs was found to be slightly larger both in PBS and human serum (Fig. 3). The final loading capacity of DOX-NBs was 64.12 mg DOX/g DOX-NBs, which corresponded to an EE of 54.18%.

### DOX Release by DOX-NBs In Vitro

**Fig. 4:**
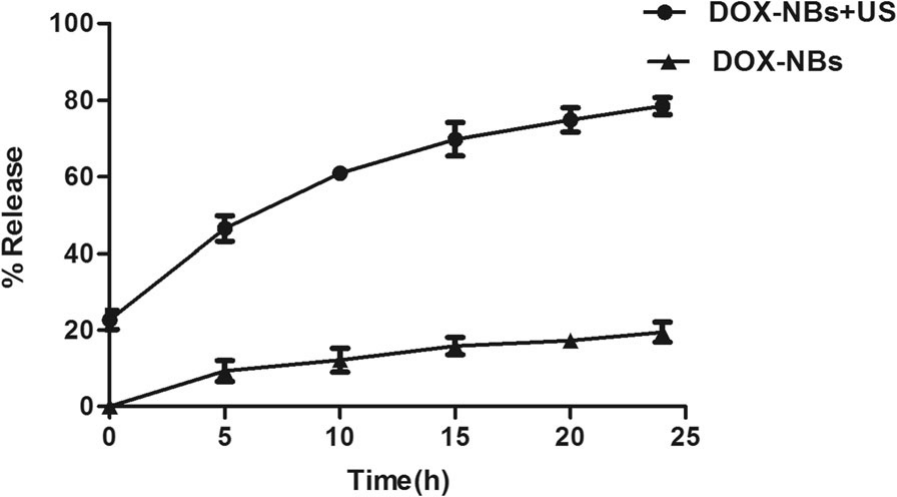
Doxorubicin release from DOX-NBs with or without ultrasonic irradiation (2 kHz, 1.0 W/cm^2^)

Figure 4 shows the in vitro release profile of DOX from DOX-NBs in PBS in the presence or absence of US treatment to assess the effects of sonication on DOX release. The amount of DOX released from DOX-NBs was significantly different between the US group and the non-US group. After 5 h, the DOX-NBs in the US group had released 46.45% of the encapsulated DOX compared to only 9.3% release in the non-US group. The non-US group released only 19.4% of DOX after 24 h. In contrast, nearly 80% of DOX was discharged in the US group. The results suggested that US irradiation may promote the release of DOX from DOX-NBs due to a cavitation effect.

### Ultrasound Stability of DOX-NBs

**Fig. 5:**
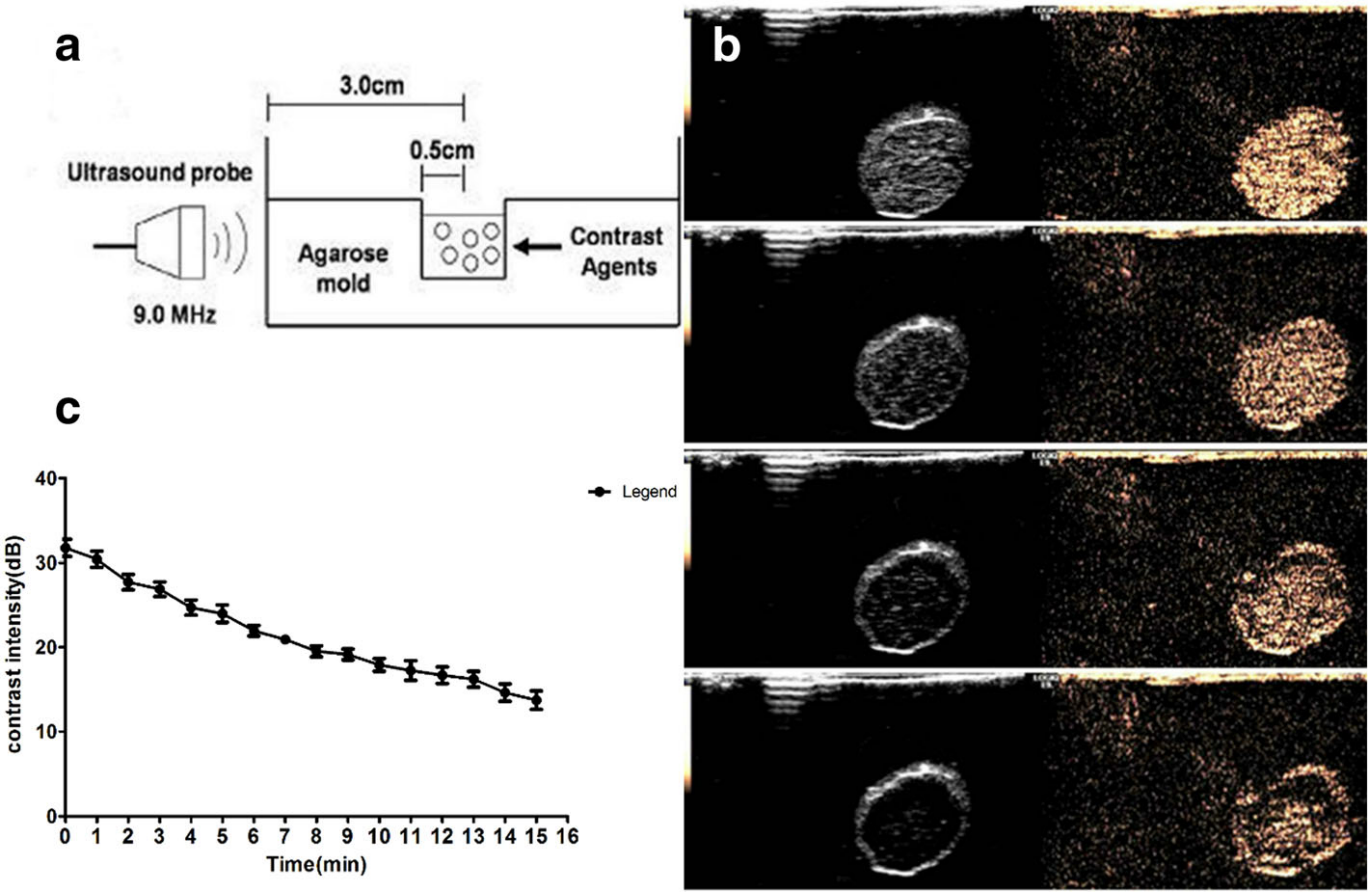
A schematic illustration of the in vitro experimental setup (**a**), ultrasound images of DOX-loaded NBs (0, 5, 10, and 15 min) using a 9.0-MHz probe (**b**) and time-intensity measurements of ultrasonic-contrast and DOX-loaded NBs (**c**)

DOX-NBs achieved ultrasound enhancement in vitro, as exhibited in Fig. 5b. The ultrasonic decibel value attenuation is shown in Fig. 5c. The results showed that DOX-NBs achieved good ultrasound enhancement, and the smooth curve demonstrates that the ultrasound attenuation process in the NB suspensions was relatively slow. This indicates that the ultrasound signal of DOX-NBs may be stable enough for the imaging and contrast enhancement.

### Biosafety of Empty NBs

**Fig. 6:**
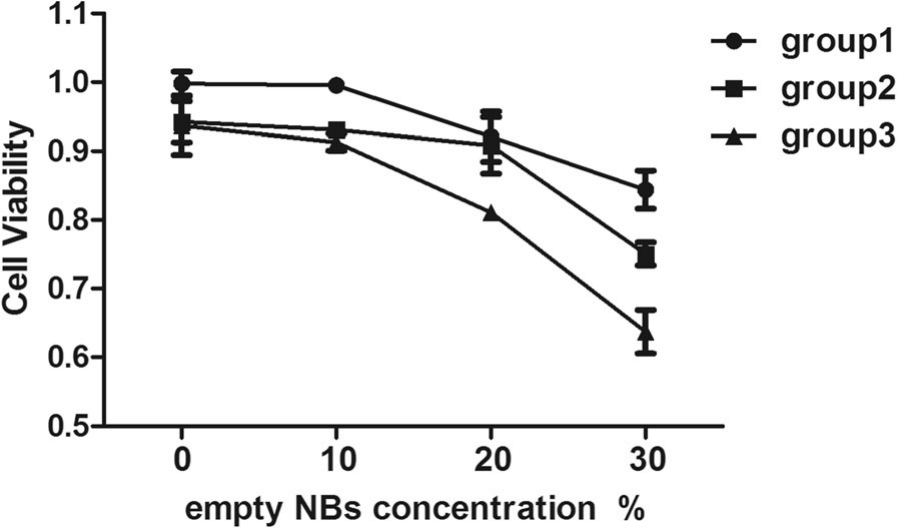
In vitro cytotoxicity of various NBs concentrations and sound intensity in MCF-7 cells

MCF-7 cell viability was measured by culturing with empty NBs (without DOX) for 24 h after ultrasound. As shown in Fig. 6, the empty NBs did not significantly affect cell viability under certain ultrasonic intensities. When using an ultrasonic intensity of 0.5 W/cm^2^ and an irradiation time of 30 s, 99.53% and > 80% of MCF-7 cells were alive in 10% and 30% NBs suspensions, respectively (group 1), and the decrease in MCF-7 cell viability was dose-dependent. In addition, ultrasonic intensity and irradiation time were two other factors that affected the viability of MCF-7 cells. In particular, when the concentration of empty NBs was 30%, the MCF-7 cells treated at 0.5 W/cm^2^ for 30 s showed a higher viability than those treated at 0.5 W/cm^2^ for 60 s or 1 W/cm^2^ for 30 s (0.84% vs. 0.75% vs. 0.63%). Therefore, an ultrasound intensity of 0.5 W/cm^2^ and an irradiation time of 30 s/60 s were used for cell uptake experiments.

### Enhancement of In Vitro DOX Delivery Mediated by DOX-NBs and Ultrasound Irradiation

**Fig. 7:**
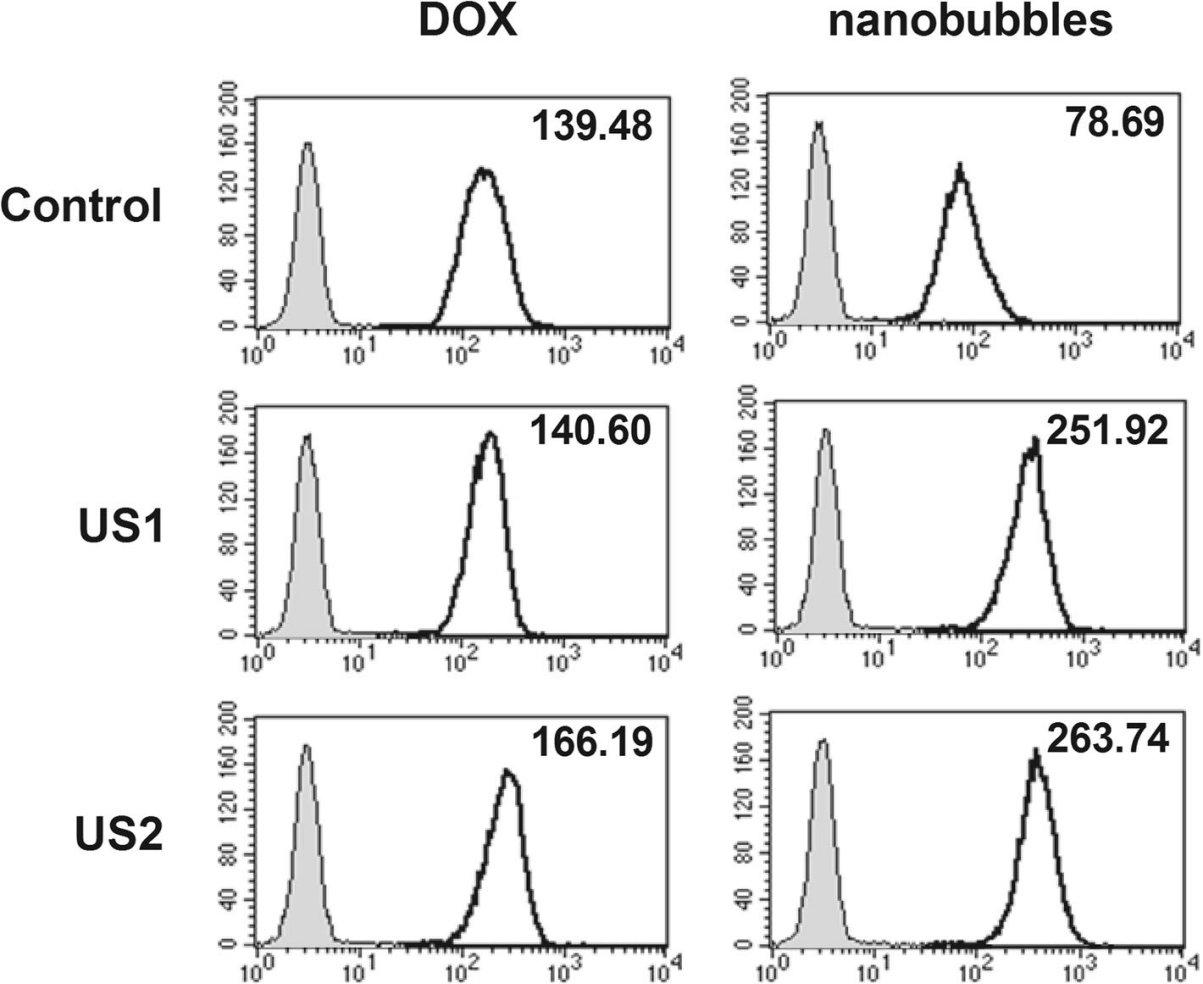
Flow cytometry analysis of DOX delivery in MCF-7 cells by DOX-loaded NBs (US1 0.5 W/cm^2^ 30 s, US2 0.5 W/cm^2^ 60 s)

The MCF-7 cells treated with DOX-NBs or free DOX (at equal DOX concentrations) were fixed, and their fluorescence intensity was measured by flow cytometry. Cells receiving no DOX treatment were used as blank controls. The cellular uptake of DOX in the DOX-NBs group was compared with that of free DOX and the control group. In Fig. 7, the mean fluorescence intensity of MCF-7 cells incubated with DOX-NBs was much lower than the autofluorescence of cells incubated with free DOX, illustrating that the encapsulation of DOX in chitosan NBs could protect cells from DOX uptake and DOX-induced injury.

However, ultrasound irradiation resulted in a marked increase in DOX uptake in MCF-7 cells incubated with DOX-NBs; DOX-NBs could deliver more DOX into MCF-7 cells with the help of ultrasonic irradiation. In contrast, the DOX uptake in MCF-7 cells incubated with free DOX was only slightly increased under ultrasonic irradiation. The results suggested that the uptake of DOX in MCF-7 cells incubated with DOX-NBs was much higher than that of the cells incubated with free DOX under ultrasonic irradiation.

In addition, the DOX uptake in MCF-7 cells incubated with DOX-NBs was slightly increased upon longer irradiation time. This may be due to an increased rupture of DOX-NBs, generating transient pores on the membranes of MCF-7 cells.

### Enhancement of DOX-Induced Tumor Cell Proliferation and Apoptosis by Ultrasound Irradiation

To investigate the anti-cancer effects of ultrasound-assisted DOX-NBs delivery, the viability of MCF-7 cells was measured using a CCK-8 assay and flow cytometry. The results show that the viability of MCF-7 cells in DOX-NBs group was higher than that in the DOX group without ultrasound. Meanwhile, the viability of MCF-7 cells in the DOX-NBs group was significantly lower than that in the DOX group with local ultrasonic irradiation. The ratio of cell viability in the DOX-NBs group (21.0 ± 2.2%, *p* < 0.01) was much higher than that in the free DOX group without ultrasonic irradiation (6.4 ± 0.7%), suggesting that as drug delivery vectors, NBs ameliorate the DOX-induced decrease of cell proliferation in blood circulation.

**Fig. 8:**
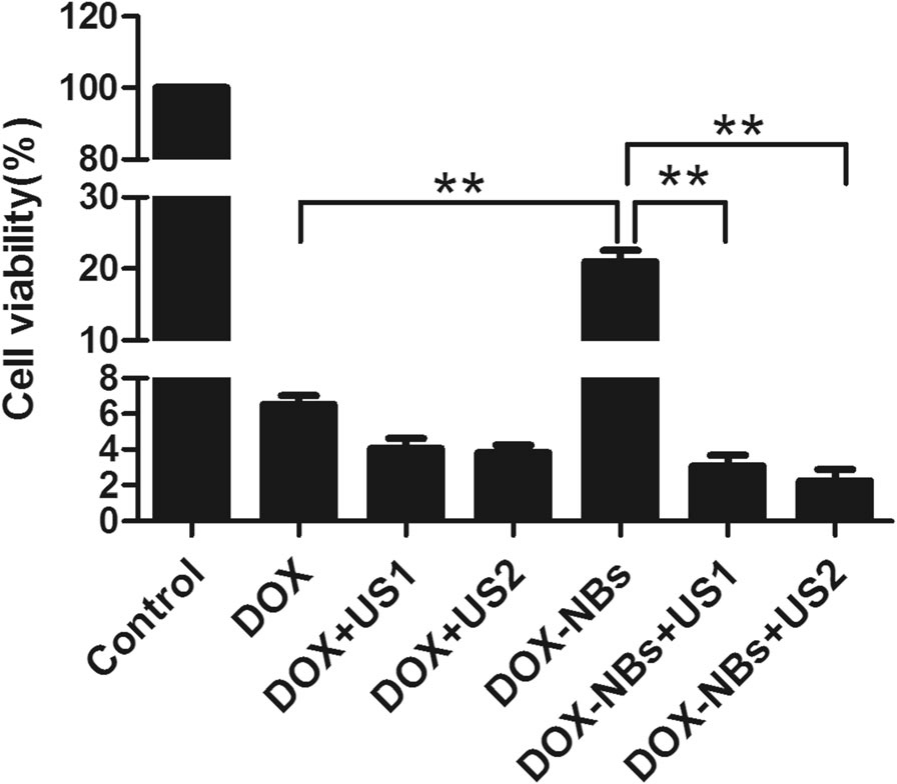
The comparison of cell viability in different groups

Moreover, the ratio of cell viability was significantly decreased in cells treated with DOX-NBs + US (3.1 ± 0.8%, 2.2 ± 0.9%) compared to those treated with DOX-NBs alone (21.0 ± 2.2%, *p* < 0.01), free DOX alone (6.4 ± 0.7%), and free DOX + ultrasound (4.1 ± 0.8%, 3.8 ± 0.6%) (Fig. 8). The data indicated that DOX-NBs + US significantly enhanced the cytotoxic effects of DOX in MCF-7 cells. The DOX-NBs + US group also demonstrated greater cytotoxicity in MCF-7 cells than the free DOX and free DOX + ultrasound groups.

The ratio of cell viability in the free DOX + ultrasound group (4.1 ± 0.8%) was lower than that in the free DOX group (6.4 ± 0.7%). Therefore, ultrasound also reduced the viability of MCF-7 cells treated with free DOX. The ratio of cell viability was 2.2 ± 0.9% when the cells were treated with DOX-NBs + ultrasound (60 s), which was lower than when treated with DOX-NBs + ultrasound (30 s) (3.1 ± 0.8%), indicating that the longer pulse length (60 s) was more efficient in DOX-NBs delivery.

Furthermore, the apoptosis of MCF-7 cells was assessed by Annexin V staining 6 h after free DOX or DOX-NB treatment, with or without ultrasound irradiation. The percentage of apoptotic MCF-7 cells in the presence of free DOX was 4.4 ± 0.9%, while a similar ratio was observed in cells treated with free DOX and ultrasound (30 s, 60 s). Delivery of the ultrasound-assisted DOX-NBs significantly increased the percentage of apoptotic cells compared to that of the free DOX group (45.7 ± 1.1% vs. 4.4 ± 0.9%, *p* < 0.01). In addition, the percentage of apoptotic cells in the DOX-NBs group without ultrasound irradiation was lower than that of the free DOX treatment group (3.2 ± 0.9% vs. 4.4 ± 0.9%). Consistent with the cell viability assay, these data indicated that ultrasound-assisted DOX-NBs delivery enhanced the anti-cancer effect of DOX.

## Discussion

Mammary cancer has attracted increasing attention due to its high incidence and mortality rates. According to a report from Globocan, mammary cancer is the most frequent cause of cancer deaths for women in less developed regions [23]. DOX is a popular anti-mammary cancer agent as it can induce DNA damage [24]. However, it can also cause severe side effects, such as cardiotoxicity, in clinical applications [25]. To overcome such toxic effects, efficient drug delivery systems that target only cancer cells are needed, thereby increasing the drug concentration at its target sites and reducing it in non-target tissues [26]. In this work, DOX-loaded biological chitosan NBs were designed, which, when used in conjunction with ultrasound, could directionally transport DOX into breast cancer cells.

Biological chitosan NBs comprised of lecithin and palmitic acid have been formulated and used for MRI/ultrasound detection, gene delivery, and oxygen delivery [13, 18, 27]. However, the drug-loading capacity and delivery efficiency of biological chitosan NBs are far from optimal. Marano et al. combined DOX-loaded glycol chitosan NBs and extracorporeal shock waves (ESWs) to enhance the antitumor activity of DOX [28]. But ESWs do not possess the imaging capability to assess the tumor size and accurately detect its location. Furthermore, although severe side effects from ESWs are rare, they may induce transient cardiac arrhythmias [29]. The metabolites of glycol including glycolate and precipitation of calcium oxalate may also cause severe metabolic acidosis [30]. Compared with ESWs, ultrasound has greater advantages due to its imaging ability, noninvasiveness, and safety.

In this study, novel DOX-NBs were prepared using perfluoropropane as the core and chitosan as the shell. Chitosan may activate macrophages and may also enhance their pro-inflammatory functions [31]. It should be noted that positively charged DOX-NBs may strongly interact with blood components, resulting in rapid clearance from the blood and suboptimal targeted accumulation at the tumor site [32]. To overcome this problem, the surface of DOX-NBs was coated with Pluronic F-68, an amphiphilic and non-ionic block copolymer formed by propylene oxide and ethylene units, which may also prevent the aggregation of nanobubbles by steric stabilization [13, 33].

Ensuring the biosafety of NBs is fundamental to their clinical application. In this study, the safety of chitosan NBs was monitored via cell viability, which showed no obvious effect on cell viability with a chitosan NB concentration of 10% and ultrasound treatment (0.5 W/cm^2^, 30 s), suggesting low cytotoxicity of chitosan NBs. Indeed, even treatment with a high dosage (30%) of chitosan NBs resulted in less than 20% observable cell death. The results showed that cell viability in chitosan NBs was much higher than that in lipid-coated nanobubbles [19], indicating that chitosan NBs were highly biocompatible with MCF-7 cells and that the cell death observed in this study may be due to the energy released by ultrasound-induced NB disruption. The high safety profile of biocompatible chitosan NBs renders them suitable for loading other drugs in the future.

Our research shows that US irradiation can effectively promote the release of DOX from DOX-NBs and subsequent cellular uptake of DOX in vitro. Exposure of cells to DOX-NBs and ultrasound resulted in near instantaneous cellular entry of DOX. The reason for this is that sonoporation is a process by which ultrasonically activated ultrasound contrast agents pulsate near biological barriers (cell membranes or endothelial layers), increasing their permeability and thereby enhancing the extravasation of external substances. In this way, drugs and genes can be delivered inside individual cells [34]. Our data showed that the cellular uptake of DOX was significantly higher in the DOX-NBs group than in the free DOX group when ultrasound-assisted delivery was applied. Meanwhile, without ultrasound, MCF-7 cells in the free DOX group showed increased DOX uptake than those in the DOX-NBs group, indicating that the chitosan NBs could reduce cellular uptake of DOX in normal tissues and protect them in the absence of ultrasonic irradiation.

Chen et al. showed that the carrier-free HCPT/DOX nanoparticles enhanced synergistic cytotoxicity against breast cancer cells in vitro [35], but they could not reduce DOX cytotoxicity and its toxicity in the circulation. A biophysical research group from Vytautas Magnus University proposed combining DOX-liposomes with microbubbles and US to enhance targeting [36]. Their results showed that the cell survival rate of DOX-liposomes decreased by 60~70% when microbubbles and ultrasound were present. By comparison, the cell survival rate of the DOX-NBs + US group in our study was 85.3% or 89.5% ((21–3.1 or 21–2.2)/21) lower than that of the DOX-NBs group. This proves that the combination of DOX-NBs and US are more effective in transporting DOX than DOX-liposomes combined with microbubbles and US. We also found that the cells in the DOX-NBs + US group showed a higher rate of apoptosis than those in the free DOX and DOX-NBs only groups. This finding was not unexpected as greater accumulation of DOX in cancer cells can increase cell death, consistent with a previous report [37].

## Conclusions

In summary, DOX-loaded biocompatible chitosan NBs were successfully prepared using a combination of biological surfactants. The prepared NBs possessed a good ability to achieve ultrasound enhancement and excellent biosafety. The in vitro results demonstrated that DOX­NBs are an innovative drug delivery system that may be useful in obtaining efficient ultrasound­assisted DOX delivery for the treatment of mammary cancer.

## Abbreviations

CCK-8: Cell Counting Kit-8
DMEM: Dulbecco’s modified Eagle’s medium
DOX: Doxorubicin hydrochloride
EE: Encapsulation efficiency
EP tubes: Eppendorf tubes
ESWs: Extracorporeal shock waves
MCF-7: Human breast adenocarcinoma cell line
NBs: Nanobubbles
US: Ultrasound
UTN/MD: Ultrasound-targeted nano/microbubble destruction
